# Mozart, Mozart Rhythm and Retrograde Mozart Effects: Evidences from Behaviours and Neurobiology Bases

**DOI:** 10.1038/srep18744

**Published:** 2016-01-21

**Authors:** Yingshou Xing, Yang Xia, Keith Kendrick, Xiuxiu Liu, Maosen Wang, Dan Wu, Hua Yang, Wei Jing, Daqing Guo, Dezhong Yao

**Affiliations:** 1 Key Laboratory for NeuroInformation of Ministry of Education, Center for Information in Medicine, University of Electronic Science and Technology of China, Chengdu, 610054; 2 School of Electronic Information Engineering, Yangtze Normal University, Fuling, Chongqing, 408100, China

## Abstract

The phenomenal finding that listening to Mozart K.448 enhances performance on spatial tasks has motivated a continuous surge in promoting music education over the past two decades. But there have been inconsistent reports in previous studies of the Mozart effect. Here conducted was a systematic study, with Mozart and retrograde Mozart music, Mozart music rhythm and pitch, behaviours and neurobiology tests, rats and humans subjects. We show that while the Mozart K.448 has positive cognitive effects, the retrograde version has a negative effect on rats’ performance in the Morris water maze test and on human subjects’ performance in the paper folding and cutting test and the pencil-and-paper maze test. Such findings are further confirmed by subsequent immunohistochemical analyses in rats on the neurogenesis and protein levels of BDNF and its receptor, TrkB. Furthermore, when the rhythm and pitch of the normal and retrograde Mozart music are manipulated independently, the learning performance of the rats in the Morris water maze test indicated that rhythm is a crucial element in producing the behavioural effects. These findings suggest that the nature of Mozart effect is the Mozart rhythm effect, and indicate that different music may have quite different to opposite effects. Further study on rhythm effect may provide clues to understand the common basis over animals from rats to humans.

Music is one of the most mysterious human phenomena, and it attracts attention from the public and scientists across disciplines worldwide^1^. Research has extensively and continuously examined the cognitive effects of music including its emotional effects on listeners and its clinic application on patients with epilepsy^2^, dementia^3^, anxiety^4^, and depression^5^. Especially, the discovery of the Mozart effect in 1993– listening to Mozart K.448 enhances spatial reasoning and memory^1^ –opened a new page for the study of music’s impact on humans. However, the following studies are not always consistent (see Supplemental Text), and the existence and reliability of Mozart effect become an intensive debate topic waiting for further systematic investigation.

Brain-derived neurotrophic factor (BDNF) and its receptor, tyrosine kinase receptor B (TrkB) are known to play a critical role in acquisition and/or consolidation of certain forms of memory in mammalian central nervous system^6^,^7^. Interestingly, the study has shown that an exposure of pregnant rats to Mozart music enhances the learning performance and increases BDNF and TrkB expression levels in newborn pups^8^. However, as music contains a multitude of elements such as rhythm, pitch, and intensity, it still remains unclear what music elements contribute to this cognitive effect. This issue speaks to a major question in music theory and practice: what is the defining character of music in such as the Mozart effect?

In music practice, the transformation of a melodic pattern, such as retrograde, reversion or retrograde reversion transformation, is widely used in Western music^9^. For example, bands have often recorded sounds or messages backwards in their songs. The Beatles used it extensively on their 1966 album *Revolver*, popularizing the effect at the time^10^. Some did it for aesthetic reasons, some for fun, and some people thought that hidden messages might be transmitted when a sound was played in reverse^11^. Now, ‘Reverse Music Player’ is easily available to the public, making the transformation of music to retrograde music just one button away. As the physical stimuli are the same for a normal and its retrograde version, do the Mozart and retrograde Mozart share the same effect?

In this study, we argue that Mozart K.448 and its retrograde version may have different effects to cognitive performance, and we guess that rhythm may be the crucial factor of Mozart effect. To confirm these hypotheses, six musical pieces were used: Mozart’s piano sonata K.448, the retrograde version of Mozart K.448, the separated rhythm, the separated pitch (Fig. 1), and the retrograde versions of rhythm and pitch. Based on these materials, a systematic study was conducted with Mozart and retrograde Mozart music on behaviours (rats, humans) and neurobiology bases (rats), normal rhythm, pitch and their retrograde versions on rats’ behaviours, respectively.

## Results

### Morris water maze test

Freely procreating rats were randomly assigned to three groups: the Mozart music group (MG), the retrograde Mozart group (RMG), and the control group (CG). Musical stimuli were played repeatedly for 12 h, from 8:00 p.m. to 8:00 a.m. daily. All acoustic interventions began on postnatal day 1 (PND 1) and continued through PND 98. The rats were subjected to the Morris water maze at three developmental stages (PND 28, PND 56, and PND 98). In the acquisition trial, which measured the escape latency to find the hidden platform, a large group difference existed in maze acquisition (*P* < 0.01). *Post hoc* tests showed that the performance significantly decreased from MG to CG then to RMG at all three of the stages. In the probe trial, the MG spent more time in the target quadrant than the CG and RMG (*P* < 0.01), and the MG exhibited less time in the opposite quadrant than the CG (*P* < 0.05; Fig. 2A).

### Human paper folding and cutting and pencil-and-paper task

As a parallel experiment, sixty undergraduate students were randomly assigned to three groups. Both the human paper folding and cutting and pencil-and-paper maze experiments clearly showed that the performance of the MG was significantly better, and the RMG exhibited a negative effect as well (Fig. 2B).

### The level of BDNF/TrkB and the neurogenesis

To explore the molecular effects above phenomenon, BDNF and TrkB protein levels were measured in the hippocampus of the rats. BDNF and TrkB levels were higher in the MG and lower in the RMG than in the CG (*P* < 0.05; Fig. 3A\C,B\D). Similar results were found in the auditory cortex (Fig. 4), but no differences were found in the parietal cortex at PND 98 (Fig. 5). Furthermore, we found that the density of DCX/BrdU double-labelled cells were greater in the MG than in the CG and RMG. There were no significant differences between the RMG and CG on PND 28 and PND 56, but the differences were significant on PND 98 (Fig. 3E–G).

### The effects of rhythm and pitch

We further examined the effects of the rhythm and pitch of the Mozart music on spatial cognition using Morris water maze task, respectively. In the acquisition trial, the performance of the Mozart rhythm group (MRG) was significantly better than the performance of the CG, while there was no significant difference between the Mozart pitch group (MPG) and the CG (Fig. 6A). In the probe trial, the rats of the MRG spent more time in the target quadrant than the CG (Fig. 6A). We then evaluated the effect of the rhythm and pitch of the retrograde Mozart music on spatial cognition. The results showed that the performance of the retrograde Mozart rhythm group (RMRG) was significantly worse than the performance of the CG, while there was no significant difference between the retrograde Mozart pitch group (RMPG) and the CG (Fig. 6B). In the probe trial, the RMRG spent less time in the target quadrant than the CG and the RMPG (Fig. 6B).

## Discussions

### The Mozart effect

There have been inconsistent reports in previous studies of the Mozart effect, due to various experimental differences across studies (see Supplemental Text), such as “litter effect”^12^ resulting from the assignment of all of the offspring from a particular mother to the same condition. To eliminate any potential litter effect, in this work, the female and male rats from the animal husbandry were allowed free procreation, and then the newborn rats were randomly assigned to different groups, resulting in groups with pups from different mothers.

In addition, previous studies compared different music, and the inconsistent results may be due to the different physical properties of the music, such as stimulus intensity, spectra and melody. In this work, except the non-music stimulus situation, we chose retrograde Mozart music (sequence in reverse order) to compare with the Mozart music to ensure that the two pieces of music had the same physical properties.

Meanwhile, we conducted behaviour experiments on both rats and human subjects. We obtained consistent behaviour results over all of our tests. Firstly, our immunohistochemical analysis showed consistent effects in the hippocampus and auditory cortex but not in the parietal cortex, suggesting that the protein expression levels of BDNF/TrkB were influenced selectively in the brain after the rats exposed to Mozart music. Note that both the BDNF and TrkB have been reported to play a vital role in mediating enduring changes of central synaptic structure and function^13^,^14^. Furthermore, we also found that newborn neurons was increased in MG and decreased in RMG. As is well known, the generation of newborn neurons in the dentate gyrus is critical for maintaining normal learning and memory processes^15^. By combining these two critical findings, our above results indicate that the improved memory scores in the spatial task (Mozart effect) are rooted in neurogenesis and enhanced BDNF/TrkB in the brain.

### Music, rhythm and species

It is well known that rhythm and pitch are the two primary dimensions of music^16^. Previous experiments have suggested that the ability to perceive rhythm may be generalized across species^17^. For instance, researchers have demonstrated that European starlings can discriminate between an isochronous rhythm and an arrhythmic sequence and maintain that discrimination even with changes in tempo^18^. Another study found that exposure to rhythmic auditory stimulus facilitates memory formation in a passive avoidance task in day-old chicks^19^. However, whether rhythm is the crucial factor for the Mozart effect has not been determined. Our results first indicated that the rhythm of Mozart music produces similar effect as Mozart music, but the pitch of Mozart music does not. These facts confirmed the crucial importance of rhythm in music and its major role in the Mozart effect.

In fact, there are questions about what the rats actually heard, whether the music was well within their hearing range, or whether artifacts in the signal might be what they are responding to. Our finding of the decisive role of rhythm in Mozart effect might have provided a reasonable explanation that rats do may not need to hear the all of the music as humans heard, they only need to get the most important rhythm to have the Mozart effect. The underlying mechanism might be that the sequence in Mozart K.448 repeating regularly every 20 to 30 seconds may trigger a strong response in the brain, because this regularity is similar to the physiological cycle^20^,^21^. And the similar effect of Mozart K.448 on both humans and rats might be due to the similar physiological rhythms evolutionally obtained from the same outside universe. Further studies on various animals would be quite valuable to understand the general role of rhythms in the evolutionary history of the nature.

### The negative effect of retrograde Mozart

Since the discovery of the Mozart effect, there has been a surge in public interest regarding the effects of music, including antenatal training. Our concern was whether a music environment is always good? Most of the reports in the literatures have focused on the beneficial effect of music^1^,^20^, but an early report also suggested that stimulation with non-rhythmic Schoenberg music (Chamber Symphony No.2) was detrimental to the performance of rats on a discrimination task^22^. In our studies, we used retrograde Mozart music as a contrast test for Mozart music. They have the same physical properties, the same frequency range and the same stimulus intensity. Such an experimental design can essentially explore the intrinsic mechanism of the effects of music. Our results showed that during early development of the auditory system, learning ability was decreased, neurogenesis was inhibited in the dentate gyrus and the level of BDNF/TrkB in the hippocampus and auditory regions were reduced in the RMG. Moreover, we found that contrary to the beneficial effect in the MG, the detrimental effect in the RMG may have been due to the retrograde rhythm (Fig. 6). In summary, both the positive effect in the MG and the negative effect in the RMG are rooted in changes of protein levels and the generation of newborn neurons, and the underlying crucial factor would be the different rhythms.

**Figure 1:**
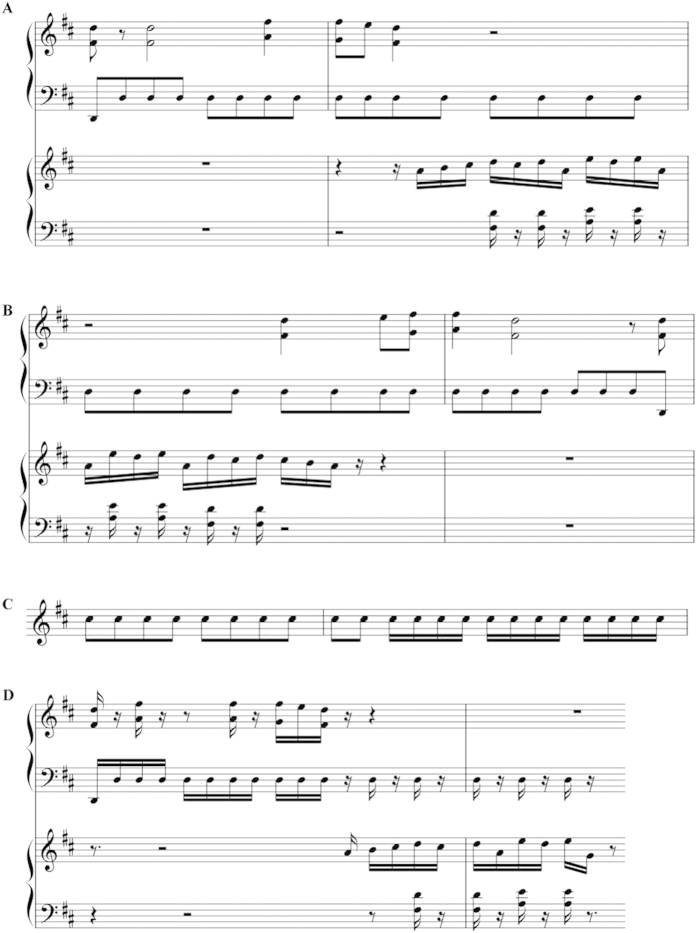
The music stimuli used in the experiments. (**A**) The fifth and sixth bars of Mozart K.448. (**B**) The retrograde version of the two bars in (**A**), which was obtained by arranging the component notes of Mozart K.448 in a reversed sequence that maintained the same physical properties as the original, except for the melodic patterns. (**C**) The rhythm of the two bars in (**A**), which preserved only the rhythm component (the pitch was changed to the same). (**D**) The pitch for the two bars in (**A**), removing the rhythm by distributing the notes uniformly in a bar.

**Figure 2:**
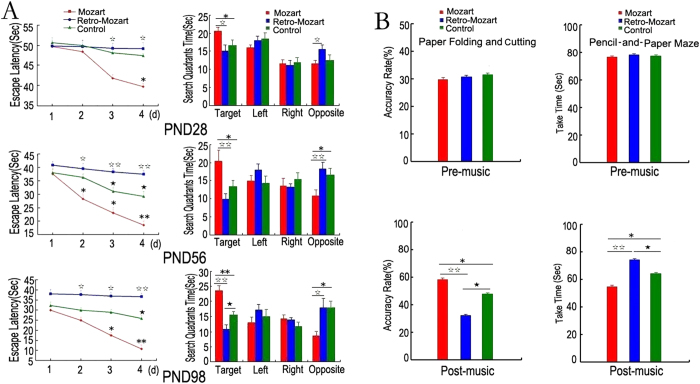
Spatial cognitive performance in developing rats and undergraduate students exposed to Mozart music and retrograde Mozart music. (**A**) Rat studies: The results from the Morris water maze test of learning and memory ability on PND 28, PND 56 and PND 98 are shown. Although all of the rats from the three different groups showed a decrease in escape latency to find the hidden platform, the rats exposed to Mozart music exhibited a faster learning curve than the other two groups. Additionally, the Mozart music group (MG) spent a significantly longer amount of time in the target quadrant compared to the other two groups. N = 15 per group. (**B**) Human studies: Both the improvement in the MG and the deterioration in the retrograde Mozart group (RMG) were significantly different from the control group (CG). Error bars represent the standard error of the mean (S.E.M.). *MG vs CG; ^★^ RMG vs CG; ^☆^ MG vs RMG. *, ^★^ and ^☆^ represent *P* < 0.05; **and ^☆☆^ represent *P* < 0.01. N = 20 per group.

**Figure 3:**
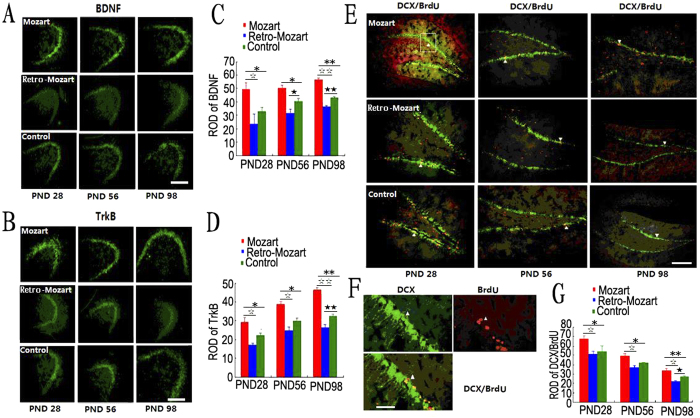
BDNF/TrkB protein expression level in the hippocampus and neurogenesis in the dentate gyrus after exposure to music. (**A–D**) The drawings in (**A–D**) show the relative optical density (ROD) of BDNF and TrkB protein levels in the hippocampus of the MG, RMG and CG. (**E**) The newborn neurons were double-labelled for DCX (green) and BrdU (red) in the dentate gyrus at three different time points (under 10 × amplification). (**F**) Images of the DCX/BrdU-expressing cell of the white pane in E (under 20 × amplification). (**G**) The statistical results of the ROD of DCX/BrdU among the groups. The data represent the mean ± S.E.M. Asterisks denote significant differences between different groups. *, ^☆^ and ^★^ represent *P* < 0.05; **, ^☆☆^ and ^★★^ represent *P* < 0.01. N = 5 per group. Scale bar: 400 μm.

**Figure 4:**
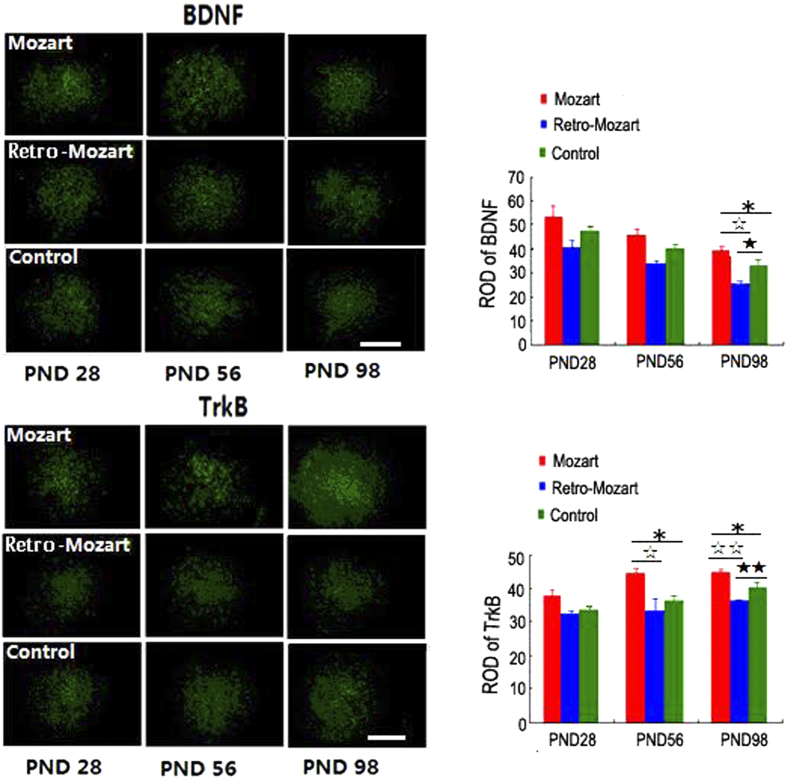
BDNF and TrkB protein expression level in the auditory cortex after exposure to music. The drawings show the ROD of BDNF and TrkB protein levels in the auditory cortex of the control rats and the rats exposed to Mozart music and retrograde Mozart music. The error bars represent the S.E.M. Asterisks denote significant differences among the groups. *, ^☆^ and ^★^ represent *P* < 0.05; ^☆^ and ^★★^ represent P < 0.01. N = 5 per group. Scale bar: 400 μm.

**Figure 5:**
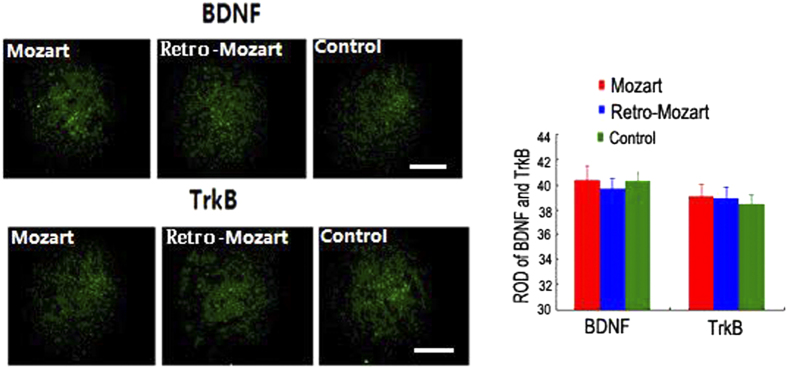
BDNF and TrkB protein expression level in the parietal cortex after exposure to music. The drawings show the ROD of BDNF and TrkB protein levels in the parietal cortex of the control rats and the rats exposed to Mozart music and retrograde Mozart music at PND 98. No significant differences were found among the groups. The error bars represent the S.E.M. N = 5 per group. Scale bar: 400 μm.

**Figure 6:**
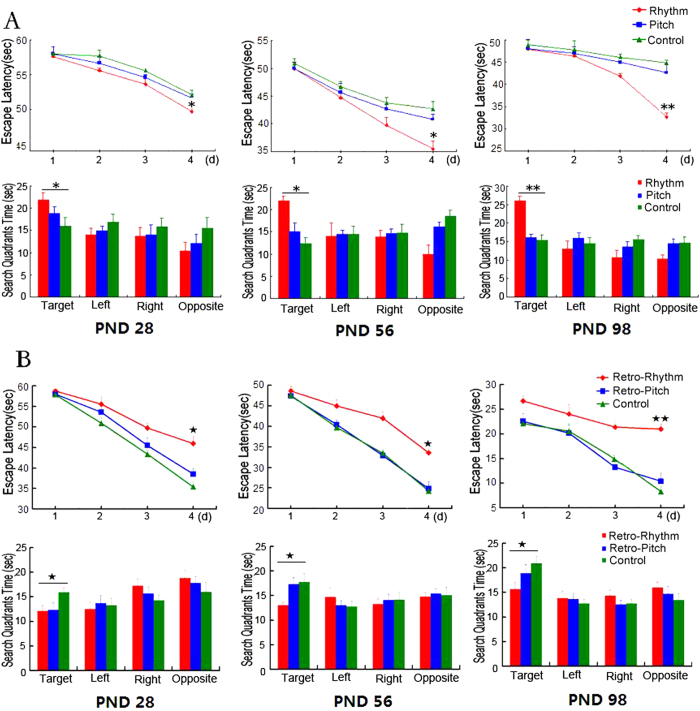
Spatial cognitive performance in developing rats exposed to the separate rhythm and pitch. (**A**) The Mozart rhythm group (MRG) exhibited a faster learning curve than the other two groups. Additionally, the time spent in the target quadrant was significantly longer in the MRG than in the other two groups. (**B**) The retrograde Mozart rhythm group (RMRG) exhibited a slower learning curve than the other two groups. In addition, the time spent in the target quadrant was significantly shorter in the RMRG than in control group. *MRG vs CG; ^★^ RMRG vs CG. *and ^★^ represent *P* < 0.05; **and ^★★^ represent *P* < 0.01. N = 15 per group.

**Figure 7:**
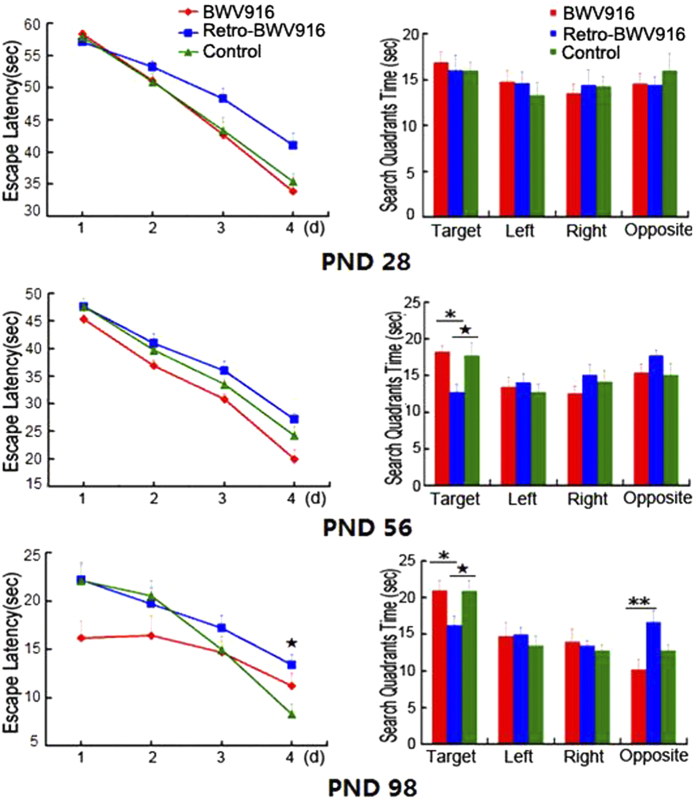
Spatial cognitive performance in developing rats exposed to Bach and retrograde Bach music. Learning and memory ability were tested using the Morris water maze on PND 28, PND 56 and PND 98. The rats exposed to retrograde Bach music exhibited a slower learning curve than the other two groups. Additionally, the time spent searching the target quadrant was significantly shorter in the retrograde Bach music group rats than in the other groups at PND 56 and PND 98. *indicates the Bach group vs the retrograde Bach group (*P* < 0.05), ^★^ indicates the retrograde Bach group vs the control group. *and ^★^ represent *P* < 0.05; **represent *P* < 0.01. N = 15 per group.

To determine whether a similar phenomenon can be also induced by other music, we chose Bach music (Bach’s Toccata in G major, BWV 916), which has been reported to have the similar effect as the Mozart music^20^, to repeat the rat experiments shown in Fig. 1. The results showed that the effects in the Bach, retrograde Bach and control groups were inferior to those in the Mozart experiment. However, the effects exhibited a similar trend. The spatial learning and memory of the retrograde Bach group (PND 98) was significantly worse than the control group (*P* < 0.05) (Fig. 7). This finding indicates that we also need to pay attention to the retrograde version of other music.

### The general experience of retrograde Mozart

**Figure 8:**
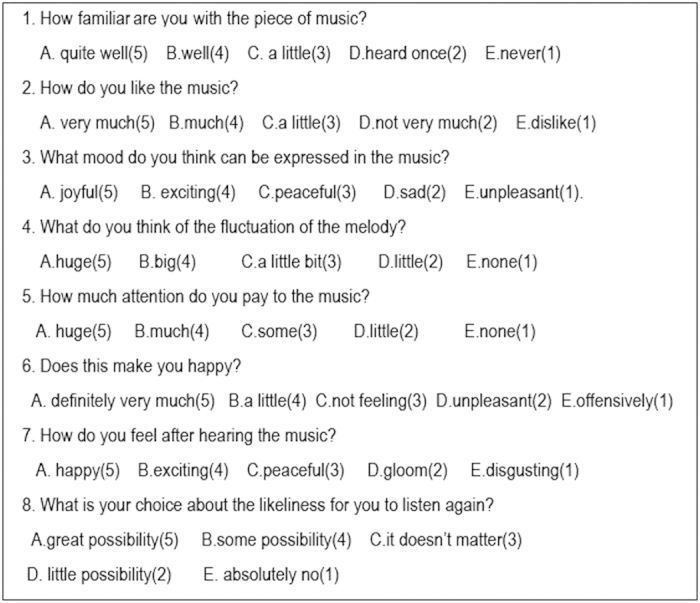
Music familiar behavioural scale. The answer of each question (**A–E**) received a score (5, 4, 3, 2 and 1, respectively); a higher score indicates a positive affection.

**Figure 9:**
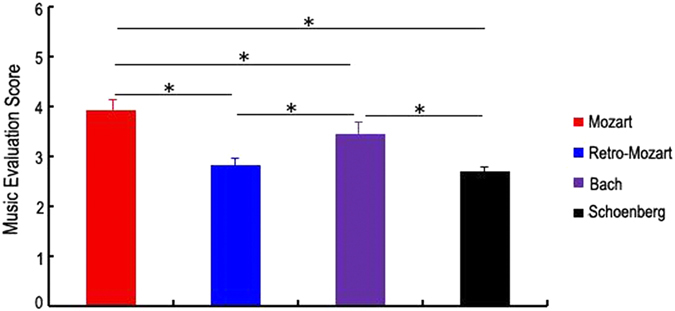
Music familiar evaluation by the undergraduates. There were significant differences between the four music groups, excluding the retrograde Mozart music and the Schoenberg music in the music familiar evaluation. The results suggest that the retrograde Mozart music and Schoenberg music had the same effect. The latter was reported to have a negative effect in a previous study. *represent *P* < 0.05. N = 15 per group.

Though retrograde transformation has been widely adopted in music, we are still curious about the experience of human beings. To address this issue, we applied a familiar behavioural scale test (Fig. 8) using Mozart, retrograde Mozart, Bach, as well as Schoenberg music (Chamber symphony No.2 Op.38-I.Adagio) which has been reported to induce a negative effect^22^. Sixty undergraduates without specialized music training took part in the experiment. The results showed that the score of the Mozart music group was significantly higher than the scores of the other groups (*P* < 0.05). The score of the Bach group was significantly higher than those of the retrograde Mozart group and Schoenberg groups (*P* < 0.05), while the scores of the retrograde Mozart group and Schoenberg groups were not significantly different (*P* > 0.05) (Fig. 9). This finding might provide a circumstantial evidence for the negative effect of the retrograde music.

Taken together, we found that Mozart K.448 and its retrograde version may have an opposite effect on spatial tasks performance, and the reason may be the retrograde change of its rhythm. Additionally, following the positive effect of Mozart and the negative effect of retrograde Mozart, there are distinct neural changes in the hippocampus and auditory cortex. These results on one side revealed the potential positive to negative effect of music around us because a ‘Reverse Music Player’ is easily accessible in current society, and some people do like retrograde one. On the other side, our findings confirmed that the Mozart and retrograde Mozart effect are due to their rhythms, and it may have provided us the link between rats effect and humans effect, and raised a general challenge problem: whether the other animals with similar physiological rhythms do have similar music effects?

## Materials and Methods

### Auditory stimuli

Six musical pieces were used in the experimental study. One was Mozart’s piano sonata K.448, which is usually used in studies of the Mozart effect. The second piece was the retrograde version of Mozart’s K.448 (retrograde Mozart), which was obtained by arranging the component notes of Mozart’s K.448 in a reversed sequence that maintained the same physical properties as the original except for the melodic patterns. The third piece was the rhythm of Mozart’s K.448, which preserved only the rhythm component (the pitch was changed to be the same throughout the piece). The fourth was the pitch component of Mozart K.448 (the rhythm was removed by distributing the notes uniformly in a bar). Figure 1 shows the material of the four music stimuli used in the experiments. The fifth and sixth stimuli were the retrograde versions of the third and fourth stimuli (omitted in Fig. 1).

### Animals and treatments

All experiments were performed according to the experimental guidelines of the University of Electronic Science and Technology of China (UESTC) and were approved by the ethics committee of the UESTC. Sprague Dawley (SD) rats born from time-mated dams (Animal Research Institute of Sichuan Province, China) at UESTC were used in the experiments. The rats were maintained on a 12-h-light/12-h-dark cycle (light on at 08:00 h) at a controlled ambient temperature (22 ± 2 °C). Food and water were made available *ad libitum*.

The environmental sound level of the control group was 65 dB (ambient noise) and the sound level of the musical group was 65–75 dB. The musical stimuli were played repeatedly for 12 h from 8:00 p.m. to 8:00 a.m. daily so that the rats would not be disturbed in their sleep. All acoustic interventions began on PND 1 and continued through PND 98.

### Morris water maze test

The hidden platform water maze task^23^,^24^ was used to study the learning and memory ability of the rats. The pool (130 cm diameter ×50 cm high) was filled with water (26 ± 1 °C) to a depth of 25 cm, and the water was made opaque with a mixture of white, thick, non-toxic milk. A circular Plexiglas platform (10 cm diameter), onto which the rat could escape, was positioned in the centre of the target quadrant. One day before each testing period, each rat was placed in the pool for 2 min as a pretraining session. The rat was then allowed to climb onto the platform where it could rest for 20 s. This procedure was repeated three times to habituate the rat to the training environment. On days 1–4 of testing, the rats performed four trials and each trial began with the rat being placed in the pool and released facing the sidewall at one of four positions (the boundaries of the four quadrants, labelled Left, Right, Target and Opposite). The time between immersion into the pool and escape onto the platform was recorded to provide a measure of spatial reference memory for each trial (four trails a day, total 16 trials for each rat).

The day after the last latency trial was completed (day 5), the platform was removed and the rats were placed in the water maze on the opposite side of the target quadrant from where the platform had previously been located. The rats were then allowed to explore the pool for 60 s (probe trials). Spatial memory ability was measured as the duration of time spent in the target quadrant (% total time, chance level = 25%).

The behavioural assessment was conducted at three time points: PND 28, PND 56 and PND 98, which were selected based on developmental stages in rats.

### Human behavioural experiment

To demonstrate that Mozart and retrograde Mozart music would produce similar effects on humans as in rats, the performed cognitive effect of humans were performed. We employed three groups of undergraduate university students without formal music training (10 males and 10 females per group), ranging in age from 18 years to 24 years (mean = 20.6 years), and each group had 20 subjects. This study was approved by the ethics committee of the University of Electronic Science and Technology of China (UESTC) and performed according to the experimental guidelines of the UESTC. Every subject provided written consent to participate in this experiment. The tests included a paper-and-pencil maze test and the paper folding and cutting test, the use of which has been well established in previous Mozart studies. Before the formal testing, 30 subjects (different from those in the formal experiment) were pretested to divide all of the test items into two sets according to the degree of difficulty. The experimental protocols were the following: on the first day, 60 subjects participated in two cognitive tests: the pencil-and-paper maze test and the paper folding and cutting test. Based on the experimental results, the 60 students were divided into three groups with approximately equivalent abilities, and each group had 20 subjects. From the second day, one of the three groups (in the same fixed classroom) listened to half an hour of Mozart music every morning (MG), continuing to the seventh day (for a total of 6 days of listening). The second group (at the same time and in the same classroom as the MG) listened to half an hour of retrograde Mozart music (RMG), continuing to the seventh day (for a total of 6 days of listening). The music was played through earphones, and subjects were blind to the type of music in both the MG and RMG; thus, they did not know what music they were going to hear. The third group of subjects remained silent in the same classroom for the same amount of time, also continuing until the seventh day (6 days of sitting in silence). On the eighth day, all three groups performed these two tasks (with different test items from the first items) at the same time as the first experiment.

### Neurogenesis and immunohistochemical procedures

Five rats from the MG, the RMG and the CG were used to detect the neurogenesis and BDNF/TrkB level. These rats had been intraperitoneally injected with 50 mg/kg 5-bromo-2’-deoxyuridine (BrdU; Sigma Chemical Co., St. Louis, MO) three times, at 4-h intervals one week before.

The rats were anesthetized and transcardially perfused with fixative (4% paraformaldehyde) at the end of each behaviour experiment (PND 28, PND 56 and PND 98). The brains were then removed and placed in the same fixative for 24 h before being transferred into a 30% sucrose solution for cryoprotection. Samples were serially sectioned in the coronal plane at 30 μm using a freezing microtome (Leica, Nussloch, Germany).

The free floating sections were processed for immunohistochemistry (IHC) as previously described^25^. Briefly, for antigen retrieval, sections were incubated with 0.25% trypsin in phosphate-buffered saline (PBS) for 5 min. Following extensive washes in PBS, the sections were blocked with 10% goat serum solution for 1 h. Primary antibodies were applied overnight at 4 °C. The sections were then washed three times with PBS and incubated for 1 h with a species-specific secondary antibody. Subsequently, the sections were extensively washed again and placed on Superfrost Plus slides. After mounting, the sections were observed and photographed using a Leica microscope equipped with a Spot^®^ digital camera.

For double BrdU/DCX immunofluorescence labelling, sections were permeabilized by incubation with 0.5% Triton X-100 in PBS for 15 min and denatured in 1 N HCl at 37 °C for 30 min. Then, the sections were washed three times in 100 mM sodium borate (pH 8.5). The remaining experimental steps were carried out as described above. However, to exclude the possibility of false labelling, primary antibodies produced in different species were co-incubated, and secondary antibodies were tested for cross reactivity in a double labelling experiment.

The following antibodies and final dilutions were used. The primary antibodies included: rabbit anti-BDNF (1:200, Santa); rabbit anti-TrkB (1:200, Abcam); mouse anti-BrdU (1:300, Cell Signalling Technology, Inc.); and goat anti-DCX (1:300, Cell Signalling Technology, Inc.). For corresponding secondary antibodies, goat anti-rabbit (1:200, Southern Biotech) donkey anti-mouse (1:200, Abcam) and donkey anti-goat (1:200, Southern Biotech) were used.

### Statistical analysis

For the behavioural tests, the escape latency during the hidden- platform acquisition training was analysed with a repeated measures analysis of variance (ANOVA) with a general linear model. Group differences in the duration of time spent in quadrants were analysed with a two-way ANOVA.

For the IHC, the area of the selected region was measured using Image-Pro Plus software (Media Cybernetics, Silver Spring, MD). The mean density of protein expressed in the area was measured automatically. Statistical analysis was performed with a one-way ANOVA.

All values are expressed as the mean ± S.E.M. (standard error of the mean), and the statistical analyses were performed using SPSS (version 16.0). *Post hoc* comparisons were performed using the Least-significant difference (LSD) method, where applicable, to provide more detail about the differences among groups. The level of statistical significance was set at *P* < 0.05.

## Additional Information

**How to cite this article**: Xing, Y. *et al.* Mozart, Mozart Rhythm and Retrograde Mozart Effects: Evidences from Behaviours and Neurobiology Bases. *Sci. Rep.* **6**, 18744; doi: 10.1038/srep18744 (2016).

## Supplementary Material

Supplementary Information
