# Supplementary material for: Mozart, Mozart Rhythm and Retrograde Mozart Effects: Evidences from Behaviours and Neurobiology Bases
Source: Sci Rep. 2016 Jan 21;6:18744. doi: 10.1038/srep18744 (PMC4726287; doi:10.1038/srep18744)
Supplement: Supplementary Information [file srep18744-s1.doc]

Mozart, Mozart Rhythm and Retrograde Mozart Effects:

From Behaviours to Neurobiology Bases

Yingshou Xing^1,2,#^, Yang Xia^1,#^, Keith Kendrick^1^, Xiuxiu Liu^1^, Maosen Wang^1^, Dan Wu^1^, Hua Yang^1^, Wei Jing^1^, Daqing Guo^1^, Dezhong Yao^1*^

**Supplemental text**

**Previous discussions on Mozart effect**

In total, 67 available publications were collected (See following Table 1). Here, only research papers were included. Meta-analysis papers, comment papers and unpublished materials were excluded. All of the studies employed spatial ability tests. P = Positive, replication of the Mozart effect; papers 1–52 all support the existence of the Mozart effect; N = Negative, failure to reproduce the Mozart effect; papers 53–67 do not support the existence of the Mozart effect; MO–NM = Mozart samples (including K.448) vs samples with a non-musical stimulus or no stimulus at all; MO–OM = Mozart samples (including K.448) vs samples with any other type of musical stimulus; OM–NM = samples with any other type of musical stimulus vs samples with a non-musical stimulus or no stimulus at all; The response (R1–R4, see below) refers to the reference that answered the studies that did not replicate the Mozart effect.

**Table 1. List of published research papers related to the Mozart effect**

| Number order | Study | P/N | Measure | Treatment condition | Response |
| --- | --- | --- | --- | --- | --- |
| 1 | Rauscher et al (1993) | P | Paper Folding and Cutting Task | MO-NM |  |
| 2 | Petsche et al. (1993) | P | EEG | MO-OM |  |
| 3 | Kenealy et al. (1994) | P | Paper Folding and Cutting Task | MO-NM |  |
| 4 | Flohr et al. (1995) | P | Visual Perspective Taking Test | MO-NM |  |
| 5 | Rauscher et al.(1995) | P | Paper Folding and Cutting Task | MO-OM |  |
| 6 | Rideout et al.(1996) | P | Paper Folding and Cutting Task | MO-NM |  |
| 7 | Wilson et al. (1997) | P | Maze Task | MO-NM |  |
| 8 | Carlson et al. (1997) | P | Delayed Response Task | MO-NM |  |
| 9 | Cash (1997) | P | [A](javascript:void(0);) [series](javascript:void(0);) [of](javascript:void(0);) Cognitive Task | OM-NM |  |
| 10 | Rideout et al. (1997) | P | Paper Folding and Cutting Task | MO-NM |  |
| 11 | Cockerton et al. (1997) | P | Alice Heim 4 test of general intelligence | OM-NM |  |
| 12 | Rauscher et al.(1997) | P | Object Assembly Task | OM-NM |  |
| 13 | Sarnthein et al. (1997) | P | EEG | MO-NM |  |
| 14 | Rauscher et al. (1998) | P | T-Maze | MO-OM |  |
| 15 | Johnson et al.(1998) | P | Spatial-Temporal Task | MO-NM |  |
| 16 | Rideout et al. (1998) | P | Paper Folding and Cutting Task | MO-NM |  |
| 17 | Rideout et al. (1998) | P | Paper Folding and Cutting Task | MO-NM |  |
| 18 | Nantais et al. (1999) | P | Paper Folding and Cutting Task | MO-NM |  |
| 19 | North et al.(1999) | P | Game | OM-NM |  |
| 20 | Shaw (2001) | P | visual-spatial task | MO-NM |  |
| 21 | Bodner et al.(2001) | P | fMRI | MO-OM |  |
| 22 | Thompson et al.(2001) | P | Paper Folding and Cutting Task | MO-OM |  |
| 23 | Toukhsati S et al. (2001) | P | Passive Avoidance Task | OM-NM |  |
| 24 | Hallam et al. (2002) | P | Arithmetic Problem | OM-NM |  |
| 25 | Hughes (2002) | P | Epileptiform Activity | MO-OM |  |
| 26 | Husain et al.(2002) | P | Paper Folding and Cutting Task | Fast MO-Slow MO |  |
| 27 | Twomey et al. (2002) | P | Stick Figures | MO-NM |  |
| 28 | Gilleta et al.( 2003) | P | Paper Folding and Cutting Task | MO-NM |  |
| 29 | Ivanov et al. (2003) | P | Paper Folding Task | MO-NM |  |
| 30 | Lints et al. (2003) | P | Paper Folding and Cutting Task | MO-OM |  |
| 31 | Sutoo et al.(2004) | P | Reduce blood pressure | MO-NM |  |
| 32 | Toukhsati et al.(2004) | P | Passive Avoidance Task | OM-NM |  |
| 33 | Aoun et al.(2005) | P | T-Maze | MO-OM |  |
| 34 | Schellenberg (2005) | P | Wechsler Intelligence Scale | MO-OM |  |
| 35 | Jones et al. (2006) | P | Paper Folding and Cutting Task | MO-NM |  |
| 36 | Chikahisa et al.(2006) | P | Cross-Maze Protein level | OM-NM |  |
| 37 | Kim et al. (2006) | P | Radial-arm Maze Test | OM-NM |  |
| 38 | Jaušovec et al. (2006) | P | Spatial Rotation Task | MO-OM |  |
| 39 | Jones et al. (2007) | P | Paper Folding and Cutting Task | MO-NM |  |
| 40 | Angelucci et al.(2007) | P | Passive Avoidance Task Protein level | OM-NM |  |
| 41 | Chikahisa et al.(2007) | P | Open field  Reduces anxiety level | OM-NM |  |
| 42 | Caldwell et al.(2007) | P | Event-related brain potentials | OM-NM |  |
| 43 | Ho et al.(2007) | P | Target Digits | MO-OM |  |
| 44 | Lahiri et al. (2007) | P | Control the seizures | MO-NM |  |
| 45 | Papoutsoglou et al. (2007) | P | Growth and Physiology | MO-NM |  |
| 46 | Xu et al. (2007) | P | GluR2 Protein Expression | OM-NM |  |
| 47 | Fukui et al. (2008) | P | Neurogenesis | MO-NM |  |
| 48 | Zhu et al. (2008) | P | ERP | MO-NM |  |
| 49 | Hyde et al.(2009) | P | [A](javascript:void(0);) [series](javascript:void(0);) [of](javascript:void(0);) Cognitive Task MRI | OM-NM |  |
| 50 | Meng et al. (2009) | P | Morris water maze  DNA microarray | MO-NM |  |
| 51 | Xu et al. (2009) | P | auditory signal-detection NR2B level | MO-NM |  |
| 52 | Pantev et al.(2011) | P | Cortical Plasticity | MO-OM |  |
| 53 | Alexander et al. (2012) | P | Reading comprehension | OM-NM |  |
| 54 | Herholz et al. (2012) | P | Review (Brain Plasticity) | MO-OM |  |
| 55 | Carstens et al. (1995) | N | Minnesota Form Board Test | OM-NM | R1 |
| 56 | Newman et al. (1995) | N | Raven's Progressive Matrices | MO-NM | R1 |
| 57 | Steele et al. (1997) | N | Backwards Digit Span Task | MO-NM | R1 |
| 58 | Steele et al (1999) | N | Paper Folding and Cutting Task | MO-NM |  |
| 59 | Chabris (1999) | N | Paper Folding and Cutting Task | MO-NM | R2 |
| 60 | Steele et al.(1999) | N | Paper Folding and Cutting Task | MO-OM |  |
| 61 | Bridgett et al. (2000) | N | Mathematics Test | MO-OM |  |
| 62 | McCutcheon (2000) | N | Object Assembly Subtest | OM-NM |  |
| 63 | McKelvie et al.(2002) | N | Paper Folding and Cutting Task | MO-OM |  |
| 64 | Steele (2003) | N | rat audiogram | MO-OM | R3 |
| 65 | Jackson et al. (2004) | N | Orientation Task | MO-OM | R4 |
| 66 | Hui (2006) | N | Pencil-and- Paper Maze | OM-NM | R4 |
| 67 | Črnčec et al.(2006) | N | Fitzgerald paper-folding test | MO-OM | R4 |

1. Rauscher F.H., Shaw, G.L. & Ky, K.N. Music and spatial task performance. *Nature* **365,**611-611 (1993).
2. Petsche, H. et al. EEG coherence and musical thinking. *Music Perception* **11,** 117-151 (1993).
3. Kenealy, P.& Monsef, A. Music and IQ tests. *The Psychologist* **7,** 346 (1994).
4. Flohr, J.W., Chesky, K.S., Persellin D. & Flohr C.M. Changes in spatial pattern ability following music listening and music vibration. Texas *Music Education Research Reports* 35-39 (1995).
5. Rauscher, F.H., Shaw, G.L.& Ky, K.N. Listening to Mozart enhances spatial temporal reasoning: towards a neurophysiological basis. *Neuroscience Letters* **185,**44-47 (1995).
6. Rideout, B.E. &Laubach, C.M. EEG correlates of enhanced spatial performance following exposure to music. *Perceptual and Motor Skills* **82,**427-432 (1996).
7. Wilson, T.S. &Brown, T.L. Reexamination of the effect of Mozart's music on spatial task performance. *Journal of Psychology* **131,**365-370 (1997).
8. Carlson, S., Rämä, P., Artchakov, D. &Linnankoski I. Effects of music and white noise on working memory performance in monkeys. *Neuroreport* **8,** 2853-2856 (1997).
9. Cash, A.H. et al. Structure of music may influence cognition. *Perceptual and motor skills* **84,**66 (1997).
10. Rideout, B.E. &Taylor, J. Enhanced spatial performance following 10 minutes exposure to music: a replication. *Perceptual and Motor Skills* **85,**112-114 (1997).
11. Cockerton, T., Moore, S. &Norman, D. Cognitive test performance and background music. *Perceptual and Motor Skills* **85,** 1435-1438 (1997).
12. Rauscher, F.H. et al. Music training causes long-term enhancement of preschool children's spatial-temporal reasoning. *Neurological research* **19(1)**, 2-8 (1997).
13. Sarnthein, J. et al. Persistent patterns of brain activity: an EEG coherence study of the positive effect of music on spatial-temporal reasoning. *Neurological research* **9(2),** 107-116 (1997).
14. Rauscher, F.H., Robinson, K.D. & Jens, J.J. Improved maze learning through early music exposure in rats. *Neurological Research* **20,**427-432 (1998).
15. Johnson, J.K., Cotman, C.W., Tasaki, C. & Shaw G.L. Enhancement of spatialtemporal reasoning after a Mozart listening condition in Alzheimer’s disease: A case study. *Neurological Research*, **20,**666-672 (1998).
16. Rideout, B.E., Dougherty, S. & Wernert L. Effects of music on spatial performance: a test of generality. *Perceptual and Motor Skills* **86,** 512-514 (1998).
17. Rideout, B.E., Fairchild, R.A. & Urban, G.E. The “Mozart effect” and skin conductance. *Paper presented at the meeting of the Eastern Psychological Association*, Boston, MA. (1998).
18. Nantais, K.M. & Schellenberg, E.G. The Mozart effect: an artifact of preference. *Psychological Science* **10,**370-373 (1999).
19. North, A.C. & Hargreaves, D.J. Music and driving game performance. *Scandinavian Journal of Psychology* **40(4),** 285-292 (1999).
20. Shaw, G.L. The Mozart effect [Letter to the editor]. *Epilepsy & Behavior* **2,**611- 613 (2001).
21. Bodner, M., Muftuler, L.T., Nalcioglu, O. & Shaw, G.L. FMRI study relevant to the Mozart effect: brain areas involved in spatial–temporal reasoning. *Neurological research* **23(7),** 683- 690 (2001).
22. Thompson, W.F. Schellenberg, E.G. &Husain, G. Arousal, mood, and the Mozart effect. *Psychological science* **12(3),** 248-251 (2001).
23. Toukhsati, S. &Rickard, N. Exposure to a rhythmic auditory stimulus facilitates memory formation for the passive avoidance task in the day-old chick. *Journal of Comparative Psychology* **115(2),** 132-139 (2001).
24. Hallam, S., Price, J. & Katsarou, G. The effects of background music on primary school pupils' task performance. *Educational studies* **28(2),** 111-122 (2002).
25. Hughes, J.R. The Mozart effect: additional data. *Epilepsy & Behavior* **3(2),** 182-184 (2002).
26. Husain, G., Thompson, W.F. & Schellenberg, E.G. Effects of musical tempo and mode on arousal, mood, and spatial abilities. *Music Perception* **20(2),** 151-171 (2002).
27. Twomey, A. &Esgate, A.The Mozart effect may only be demonstrable in nonmusicians. *Perceptual and Motor Skills* **95,**1013-1026 (2002).
28. Gilleta, K.S., Vrbancic, M.I., Elias L.J. &Saucier D.M. A Mozart effect for women on a mental rotations task. *Perceptual and Motor Skills* **96,** 1086-1092 (2003).
29. Ivanov, V.K. &Geake, J.G. The Mozart effect and primary school children. *Psychology of Music* **31,**405-413 (2003).
30. Lints, A. &Gadbois, S. Is listening to Mozart the only way to enhance spatial reasoning? *Perceptual and Motor Skills* **97,**1163-1174 (2003).
31. Sutoo, D.e. &Akiyama, K. Music improves dopaminergic neurotransmission: demonstration based on the effect of music on blood pressure regulation. *Brain research* **1016(2),** 255-262 (2004).
32. Toukhsati, S. &Rickard, N. Variations in Intensity and Frequency Moderate the Facilitative Effects of a Complex Rhythm Stimulus on Long-Term Memory Consolidation in the Day-Old Chick. *Journal of Comparative Psychology* **118(1)**, 65-70 (2004).
33. Aoun, P., Jones, T., Shaw, G.L. &Bodner M. Long-term enhancement of maze learning in mice via a generalized Mozart effect. *Neurological research* **27(8),** 791-796 (2005).
34. Schellenberg, E.G. Music and cognitive abilities. *Current Directions in Psychological Science* **14(6),** 317-320 (2005).
35. Jones, M.H., West, S.D. &Estell, D.B. The Mozart effect: Arousal, preference, and spatial performance. *Psychology of Aesthetics, Creativity, and the Arts* **1,**26-32 (2006).
36. Chikahisa, S. et al. Exposure to music in the perinatal period enhances learning performance and alters BDNF/TrkB signaling in mice as adults. *Behavioural brain research* **169(2),** 312- 319 (2006).
37. Kim, H. et al. Influence of prenatal noise and music on the spatial memory and neurogenesis in the hippocampus of developing rats. *Brain and Development* **28(2),** 109-114 (2006).
38. Jaušovec, N., Jaušovec, K. &Gerlič, I. The influence of Mozart’s music on brain activity in the process of learning. *Clinical Neurophysiology* **117(12),** 2703-2714 (2006).
39. Jones, M.H. &Estell, D.B. Exploring the Mozart effect among high school students. *Psychology of Aesthetics, Creativity, and the Arts* **1,**219-224 (2007).
40. Angelucci, F., Fiore, M., Ricci, E., Padua, L., Sabino, A. &Tonali, P.A. Investigating the neurobiology of music: brain-derived neurotrophic factor modulation in the hippocampus of young adult mice. *Behavioural pharmacology* **18(5-6),** 491-496 (2007).
41. Chikahisa, S., Sano, A., Kitaoka, K., Miyamoto, K-i. &Sei H. Anxiolytic effect of music depends on ovarian steroid in female mice. *Behavioural brain research* **179(1),** 50-59 (2007).
42. Caldwell, G.N. &Riby, L.M. The effects of music exposure and own genre preference on conscious and unconscious cognitive processes: a pilot ERP study. *Consciousness and Cognition* **16(4),** 992-996 (2007).
43. Ho, C., Mason, O. &Spence, C. An investigation into the temporal dimension of the Mozart effect: Evidence from the attentional blink task. *Acta psychologica* **125(1),** 117-128 (2007).
44. Lahiri, N. &Duncan, J.S. The Mozart effect: encore. *Epilepsy & Behavior* **11(1),** 152-153 (2007).
45. Papoutsoglou, S. et al. Effect of Mozart's music (Romanze-Andante of “Eine Kleine Nacht Musik”, sol major, K525) stimulus on common carp physiology under different light conditions. *Aquacultural engineering* **36(1),** 61-72 (2007).
46. Xu, F., Cai, R., Xu, J., Zhang, J. &Sun, X. Early music exposure modifies GluR2 protein expression in rat auditory cortex and anterior cingulate cortex. *Neuroscience letters* **420(2),**179-183 (2007).
47. Fukui, H. &Toyoshima, K. Music facilitate the neurogenesis, regeneration and repair of neurons. *Medical hypotheses* **71(5),** 765-769 (2008).
48. Zhu, W. et al. The influence of Mozart's sonata K. 448 on visual attention: An ERPs study. *Neuroscience letters* **434(1),**35-40 (2008).
49. Hyde, K.L. et al. Musical training shapes structural brain development. *The Journal of Neuroscience* **29(10),** 3019-3025 (2009).
50. Meng, B., Zhu, S., Li, S., Zeng, Q. &Mei, B. Global view of the mechanisms of improved learning and memory capability in mice with music-exposure by microarray. *Brain research bulletin* **80(1),** 36-44 (2009).
51. Xu, J., Yu, L., Cai, R., Zhang, J. & Sun, X. Early auditory enrichment with music enhances auditory discrimination learning and alters NR2B protein expression in rat auditory cortex. *Behavioural brain research* **196(1),** 49-54 (2009).
52. Pantev, C. & Herholz, S.C., Plasticity of the human auditory cortex related to musical training. *Neuroscience & Biobehavioral Reviews* **35(10),** 2140-2154 (2011).
53. Alexander, J., Firouzbakht, P., Glennon, L. &Lang, M. Effects of music type on reading comprehension performance and other physiological factors. *Journal of Advanced Student Science (JASS)* **1(1),** 1-11 (2012).
54. Herholz, S.C. & Zatorre, R.J. Musical training as a framework for brain plasticity: behavior, function, and structure. *Neuron* **76(3),** 486-502 (2012).
55. Carstens, C.B., Huskins, E. &Hounshell, G.W. Listening to Mozart may not enhance performance on the revised Minnesota paper form board test. *Psychological Reports* **77,**111-114 (1995).
56. Newman, J. et al. An Experimental Test of' the Mozart Effect': Does Listening to His Music Improve Spatial Ability? *Perceptual and Motor Skills* **81,** 1379-1387 (1995).
57. Steele, K.M., Ball, T.N. &Runk, R. Listening to Mozart does not enhance backwards digit span performance. *Perceptual and motor skills* **84(3c),** 1179-1184 (1997).
58. Steele, K.M., Brown, J.D. &Stoecker, J.A. Failure to confirm the Rauscher and Shaw description of recovery of the Mozart effect. *Perceptual and Motor Skills* **88,**843-848 (1999).
59. Chabris, C.F. Prelude or requiem for the Mozart effect? *Nature* **400**,826-827 (1999).
60. Steele, K.M., Bass, K.E. &Crook M.D. The mystery of the Mozart effect: Failure to replicate. *Psychological Science* **10(4),** 366-369 (1999).
61. Bridgett, D.J. &Cuevas, J. Effects of listening to Mozart and Bach on the performance of a mathematical test. *Perceptual and Motor Skills* **90(3c),** 1171-1175 (2000).
62. McCutcheon, L.E. Another failure to generalize the Mozart effect. *Psychological Reports* **87(1),** 325-330 (2000).
63. McKelvie, P. &Low, J. Listening to Mozart does not improve children's spatial ability: Final curtains for the Mozart effect. *British Journal of Developmental Psychology* **20,**241-258 (2002).
64. Steele, K.M. Do rats show a Mozart effect? *Music Perception* **21,**251-265 (2003).
65. Jackson, C.S. &Tlauka, M. Route-learning and the Mozart effect. Psychology of Music **32,**213-220 (2004).
66. Hui, K. Mozart effect in preschool children? Early Child Development and Care **176,**411-419 (2006).
67. Črnčec, R., Wilson, S.J. &Prior, M. No evidence for the Mozart effect in children. *Music Perception* **23(4),** 305-318 (2006).

**R1:** **Rauscher, F.H. & Shaw, G.L. Key components of the Mozart effect. *Perceptual and motor skills* 86(3), 835-841(1998).**

“We suggest that two components of spatial components―are essential for the Mozart effect. Studies have shown tasks which require these subskills of spatial ability, i.e., paper folding and maze tasks, were subject to enhancement following exposure to music, whereas tasks which do not require these subskills, i.e., matrices tasks, which lack both components, the digit span task, which lacks spatial imagery, and the paper formboard task, which lacks temporal ordering, were not enhanced.”We agree that researchers should use a suitable task to measure spatial reasoning, and the task used to measure spatial reasoning should test the mental rotation ability of the subjects under the physical model condition.

**R2: Rauscher, F.H. reply: Prelude or requiem for the ‘Mozart effect’? *Nature* 400(6747), 827-828 (1999).**

“Steele et al. find no Mozart effect in three differently designed studies. Not one design replicated the original reports, and they introduced several methodological concerns. For example, spatial–temporal task performance varies widely between individuals, making randomization an inefficient way to ensure uniform before-treatment task proficiency. What measures were taken by the two studies using between-subjects designs to tackle this? Was testing done blind, as in other replications?”Taken together, there are some problems in the experimental procedure, such as control of differences in the ability of the individual subjects, control of experimenter effect, etc.

**R3: Frances, F.H.** **The Mozart effect in rats: Response to Steele. *Music Perception* 123(5), 447-453 (2006).**

(1) Steele reports ‘a count of the amount of each note’ in the first movement of K.448, which leads him to assert that rats would not have heard 1,913 of the 2,790 (69%) notes in the first movement. Our own analysis disagrees considerably with Steele’s estimate of the total number of notes in the Mozart sonata.”

(2)“To perform the note-count analysis, we acquired a Musical Instrument Digital Interface (MIDI) version of the first movement of K.448 (Classical Music Midi Page, n.d.) and compared it and a written score (Sheet Music Archive, n.d.) against the recorded version played to the rats (Mozart, 1781) to assure they were equivalent. We then used MIDINote (Nagler, n.d.) to produce a list of the notes in the MIDI performance. Our count produced 9,363 notes, not 2,790 notes as Steele reported. The discrepancy between Steele’s note count and our own is inconsequential using Steele’s threshold of 500 Hz because it did not substantially alter the proportion of notes that the rats may have potentially heard. However, the proportion of the 9,363 notes with a fundamental frequency above 250 Hz was 57% (5,329 notes).”

(3)“Just as in research with human infants, one cannot really “know” what rats hear when exposed to the first movement of the Mozart piano sonata K.448. The critical point, however, is that Rauscher, Robinson, and Jens (*Rauscher, F.H., Robinson, K.D. & Jens, J.J. Improved maze learning through early music exposure in rats. Neurological research* ***20(5),*** *427-32 (1998)*) clearly demonstrate that some aspect or aspects of that sensory stimulus influenced the rats’ maze running performance in a distinctly different manner than did white noise, a Phillip Glass composition, or silence. This finding must be explained. Although rats certainly hear a higher range of frequencies than do humans, studies designed to test auditory sensitivity generally employ sine-wave stimuli.”

Taking these studies together, we agree that exposing rats to music can change the physiological state of their brain to a certain extent. In animals, the Mozart effect has a unique neural physiological and psychological mechanism, and the mechanism of the rat Mozart effect may be different from the mechanism of the effect in humans, as proposed in arousal theory *(Thompson, W.F., Schellenberg, E.G.& Husain G. Arousal, mood, and the Mozart effect. Psychological science* ***12(3),*** *248-251(2001))*.

**R4 (about papers 65-67):**

(1) Paper 65: Although the experiment applied the maze via computer simulation, there were many differences from the paper-and-pencil maze; the main difference was that the computer maze focuses on the judgement of orientation and lacked spatial information overall. That is to say, the task lacked spatial imagery, which produces the Mozart effect.

(2) Paper 66: There were two defects in the experimental design. First, after grouping, each group had only a small number of subjects (7 subjects). Second, the control group consisted of the game condition instead of a silent state, which affects the experimental results.

(3) Paper 67: The study had one limitation. Although the experiment adopted a mixed design, a verbal interference task was not inserted between the pretest and the experimental treatment; therefore, an effect in the pretest may be contained in the process of the experiment. In other words, the effect may be continued in the Mozart music condition and hinder the improvement of spatial reasoning, causing there to be no significant differences in test scores between the Mozart group and the control group with silent stimulation *(Rauscher, F.H. & Shaw, G.L. Key components of the Mozart effect. Perceptual and motor skills* ***86(3)****,835-41(1998)).*

In addition to the R1-R4 above, the choice of music is also very important. Not all music can produce the Mozart effect, according to Rauscher. Rauscher et al believe that music that has a complex structure, like the Mozart sonata, can improve spatial reasoning scores *(Rauscher, F.H., Shaw, G. L. Ky, K.N.* *Music and spatial task performance. Nature* ***365****, 611-611(1993)).*

In conclusion, in our opinion, the Mozart effect is object it can be reproduced if you adopt the same stimuli and use the same spatial capacity measure. However, people hope the effect will be more widespread, for example, to be true of other classical music and to affect other measures including IQ. Nevertheless, the results to date are not consistent regarding an extended range of this effect.

In this work, we simply adopted the original Mozart K.448 and the behaviour measurement adopted by Rauscher et al. As the ‘Reverse Music Player’ is easily accessible in current society, our concern is about the effect of retrograde Mozart compared to the original Mozart effect. Furthermore, we added an immunohistochemical analysis on neurogenesis and BDNF/TrkB protein level to investigate the changes in the brain.
